# Urban-like night illumination reduces melatonin release in European blackbirds (*Turdus merula*): implications of city life for biological time-keeping of songbirds

**DOI:** 10.1186/1742-9994-10-60

**Published:** 2013-10-03

**Authors:** Davide M Dominoni, Wolfgang Goymann, Barbara Helm, Jesko Partecke

**Affiliations:** 1 Department of Migration and Immuno-ecology, Max Planck Institute for Ornithology, Radolfzell, Germany; 2 Department of Biology, University of Konstanz, Konstanz, Germany; 3 Institute of Biodiversity, Animal Health and Comparative Medicine, University of Glasgow, Glasgow, UK; 4 Department of Behavioural Neurobiology, Max Planck Institute for Ornithology, Seewiesen, Germany

**Keywords:** Light-at-night, Melatonin, Urbanization, Locomotor activity, GnIH, Circadian rhythms, Timing of reproduction, Birds

## Abstract

**Introduction:**

Artificial light-at-night is known to affect a broad array of behaviours and physiological processes. In urbanized bird species, light-at-night advances important biological rhythms such as daily cycles of activity/rest and timing of reproduction, but our knowledge of the underlying physiological mechanisms is limited. Given its role as chronobiological signal, melatonin is a strong candidate for mediating the effects of light-at-night.

**Results:**

We exposed urban and rural European blackbirds (*Turdus merula*) to two light treatments equal in photoperiod but with different light intensities at night. The control group was exposed to 0.0001 lux (almost darkness), while the experimental group was exposed to 0.3 lux at night, simulating conditions recorded previously on free-living urban blackbirds. We obtained diel profiles of plasma melatonin for all birds in summer (July) and winter (January), while simultaneously recording locomotor activity. Daily patterns of melatonin concentrations were clearly affected by light-at-night in both seasons. In winter, melatonin concentrations of light-at-night birds were lower in the early and late night than in those of birds kept in darkness. In summer, melatonin concentrations of the light-at-night birds were lower through all night compared to birds kept in darkness. Locomotor activity in light-at-night birds was overall higher than in control individuals, both during the day and at night, and it increased sharply before dawn. In winter, the amount of activity before dawn in the light-at-night group correlated with changes in melatonin from midnight to late night: the greater the decrease in melatonin, the greater the amount of pre-dawn activity. Urban and rural birds responded similarly to light-at-night with respect to melatonin, but differed in their behaviour, with rural birds showing more locomotor activity than urban counterparts.

**Conclusions:**

This study points to reduced melatonin release at night as a potential physiological mechanism underlying the advanced onset of morning activity of urbanized birds. Based on the pattern of melatonin secretion, we suggest that birds responded to light-at-night as if they were exposed to a longer day than birds kept under dark nights.

## Introduction

In 1879 the first commercially produced light bulb illuminated the streets of New York City [1]. More than a century later, the use of artificial light is now widespread and has lead to a dramatic change in the lifestyle of billions of people. This surely brought economic and social benefits, but in the last decades the negative effects of the exposure to light-at-night have started to become apparent, especially because of the potential implications for humans [2,3]. While the effects of artificial lighting on human health and economy have stimulated a great body of research [4,5], the understanding of the potential consequences of light pollution for wildlife had been limited to few and sparse examples [6-8]. However, in the last decade new interest in this topic has arisen [9].

In birds, two of the most frequently reported effects of light-at-night are an advanced onset of activity in the morning [10-12] and an advanced seasonal onset of reproduction [10,12,13]. These effects are not surprising given the importance of light as crucial information for the regulation of both daily and seasonal processes in avian species [14,15]. It has been suggested that light-at-night might alter daylength detection [13,16,17], but we currently do not understand the physiological mechanisms that are involved in this potential process. Because of its central role in the vertebrate circadian system and its involvement in seasonal processes of many different taxa, the hormone melatonin is a strong candidate to mediate effects of light-at-night on daily and seasonal rhythms. In most vertebrates melatonin is primarily secreted by the pineal gland in a rhythmic fashion: it is released at night and suppressed by daylight [18]. Melatonin is a potent signal of daily timing and its periodic administration is able to synchronize the endogenous circadian clock even after removal of the pineal gland [19-21]. In addition, many studies have confirmed that the timing of daily rhythms such as sleep, body temperature and cortisol, can be regulated by exogenous melatonin [21-24]. Two major characteristics of the melatonin rhythm have been studied extensively, particularly in mammals and birds: the duration of nocturnal melatonin release and its diel amplitude.

The duration of melatonin secretion at night is considered to provide a reliable physiological signal of darkness, and by implication, daylength [25,26]. In mammals this signal is used to encode both time of day and time of year [27,28]. In many songbird species, daily melatonin rhythms are necessary for the persistence of circadian rhythms in constant conditions and for the synchronization of the circadian clock to the light/dark cycle [29,30], but the involvement of melatonin in annual timing has traditionally been questioned [14,31-33]. In the last 15 years, however, laboratory studies have shown that the duration of melatonin secretion can be linked to the i) seasonal variation in immune function ([34], but see [35]), ii) seasonal plasticity in song control nuclei [36] and iii) regulation of gonadotropin-inhibitory hormone (GnIH) release [37]. In addition, new data from the field have revealed that a manipulation of the melatonin signal through the continuous administration of melatonin via silastic implants can delay the time of egg-laying in a wild songbird, the great tit (*Parus major*) [38].

The diel amplitude of melatonin concentration shows great variation within and across taxa. The factor studied most often for its involvement in the modulation of melatonin release at night is light intensity. Indeed, in mammals the intensity of the illumination at night determines the suppression of melatonin release. Light intensities of 300 lux have been shown to reduce melatonin concentration [39-42] and to delay the onset of melatonin secretion at night [43,44]. In addition, exposure to longer light pulses at night is more suppressive than to short light pulses [40,41]. In songbirds, both constant light exposure and pinealectomy usually suppress the daily rhythm in melatonin [29,45,46], but we still have a poor understanding of the level of light intensity at night that alters the amplitude of the melatonin rhythm, and of the additional role of the photic contrast in light intensities between subjective day and night [47].

Melatonin is also known to be functionally associated with locomotor activity in a number of species [20,22,48-51]. Given the reported changes of activity patterns in animals which colonize new environments, melatonin patterns could thus also be modified, in particular if animals are exposed to artificial light-at-night. We therefore set up an experiment with the goal to elucidate whether light-at-night can alter the diel release of melatonin, and how this change may modify daily cycles of activity. We experimentally exposed birds that were collected from urban and rural areas to light-at-night of very low intensity, based on levels obtained with light loggers carried by free-ranging conspecifics [13]. Specifically, we asked: i) whether low light intensity at night, representative of exposure of birds in urban areas, is able to alter the nocturnal production of melatonin; ii) whether this change in melatonin secretion, if present, can be related to shift in timing of activity in the morning; iii) whether possible effects of light-at-night on the melatonin rhythm depend on photoperiod (i.e. differ between winter and summer), and iv) whether birds of urban origin differed from those of rural origin. We used the European blackbird (*Turdus merula*) as a model species. Blackbirds are widespread in Europe and breed successfully in cities [52], where they show an earlier onset of morning activity [53,54] and earlier timing of reproduction [55] compared to forest conspecifics. Light-at-night has recently been associated with such shifts in daily and seasonal timing in this species [13], but knowledge of the mechanisms involved is lacking.

## Results

**Figure 1:**
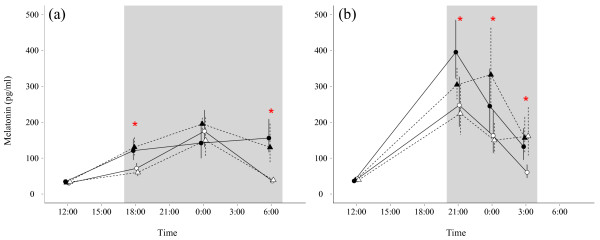
**Variation in diel melatonin concentration in winter and summer.** Melatonin was measured in winter **(a)** at 6:00, 12:00, 18:00, and 24:00, and in summer **(b)** at 3:00, 12:00, 21:00 and 24:00. The mid-night and mid-day sampling times were the same in both seasons, but we modified the evening and morning samplings in order to keep the time distance to the respective twilights equal. X-axis represents the time of day at which melatonin samples were taken, but we staggered the symbols to facilitate visualization. Y-axis represents back transformed log melatonin concentration. Black symbols depict birds under dark nights (circles, rural (N = 10); triangles, urban (N = 9)), white symbols depict birds under light-at-night of 0.3 lux (circles, rural (N = 10); triangles, urban (N = 10)). Error bars (solid, rural; dashed, urban) represent back-transformed SEM. Red asterisks depict significant treatment effect (light-at-night vs. dark night) at specific times of day, on birds from the two populations combined.

Plasma melatonin concentration differed significantly between seasons (LMM, t = −5.17, pMCMC < 0.001), with lower mean levels in winter than in summer (back-transformed log concentration: winter: mean = 75.86, lower = 70.14, upper = 82.03; summer: mean = 128.82, lower = 117.49, upper = 141.25; Figure 1). During the long winter nights, plasma melatonin concentrations were significantly lower in the light-at-night group than in the dark-night group in the evening and in the morning, whereas both groups did not differ from each other at midnight (LMM, treatment*time of day interaction, t = −3.2, pMCMC < 0.001; Figure 1a, Table 1 and Additional file 1: S2). Conversely, we did not detect a significant difference between populations (t = −0.1, pMCMC = 0.955, Table 1). In summer, under shorter nights, birds under light-at-night expressed significantly lower levels of melatonin throughout the night than birds in the dark-night group (LMM, t = −1.9, pMCMC = 0.038; Table 1 and Figure 1b). Similar to winter, there were no main or interactive effects of the birds’ origin on melatonin concentrations in summer (LMM, t = 0.74, pMCMC = 0.406; Table 1).

**Table 1:** Variation in plasma melatonin concentration

	**Winter**				**Summer**			
**Parameters**	**Estimate**	**Lower 95%**	**Upper 95%**	**pMCMC**	**Estimate**	**Lower 95%**	**Upper 95%**	**pMCMC**
Intercept	1.15	0.44	1.88	0.002	1.01	0.2	1.81	0.015
treatment	0.3	−0.26	0.88	0.297	−0.32	−0.62	−0.02	**0.038**
origin	−0.01	−0.25	0.24	0.955	0.12	−0.17	0.42	0.406
time of day	2.76	2.16	3.35	**< 0.001**	3.5	2.79	4.2	**< 0.001**
time of day^2^	−0.46	−0.57	−0.34	**< 0.001**	−0.64	−0.78	−0.5	**< 0.001**
treatment * time of day	−0.33	−0.54	−0.12	**0.002**				

Models are LMMs. Reference levels: treatment = control group, origin = rural birds. For each parameter we show the estimated mean, the lower and upper 95% CI and the p-value calculated based on MCMC (pMCMC). Non-significant interactions were sequentially removed (empty cells). Significant results are printed in bold.

The analysis of individual melatonin amplitude (measured as the difference between the diel maximum and minimum in an individual) gave similar results. Birds showed a 14.5% reduction in melatonin amplitude in winter compared to summer, and this difference was significant (LMM, t = −4.63, pMCMC < 0.001, Additional file 1: Table S1). Birds exposed to light-at-night showed a nearly-significant tendency to have a reduced melatonin amplitude compared to birds under dark-nights, in both summer (−3.7%) and winter (−13.13%) (LMM, t = 1.65, pMCMC = 0.052, Additional file 1: Table S1). There was no effect of rural or urban origin on melatonin amplitude (LMM, t = 0.04, pMCMC = 0.96, Additional file 1: Table S1), nor were interactions between the factors significant.

**Figure 2:**
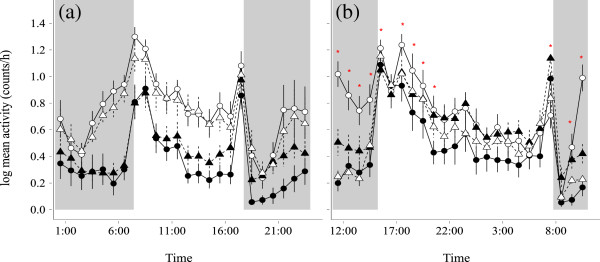
**Variation in daily cycles of locomotor activity.** We used one week of activity data before the start of the melatonin sampling, in both winter **(a)** and summer **(b)**. X-axis represents time of day, Y-axis represents log-transformed mean (± SEM) activity level (counts per hour). For sample sizes and meaning of colours and symbols see Figure 1. In winter we detected treatment effects over the entire day, in summer only at specific hours (depicted by red asterisks).

The light treatment significantly increased average locomotor activity in both seasons. In winter, activity increased at all hours of day (LMM, pMCMC < 0.001, Table 2; Figure 2a and Additional file 1: Figure S1a), without a significant effect of origin (LMM, pMCMC = 0.629, Table 2). In summer the difference in locomotor activity levels depended on treatment, origin and the time of day (three-way interaction time of day*treatment*origin: LMM, pMCMC = 0.007, Table 2 and Additional file 1: Figure S1b). Average activity under light-at-night was significantly higher than under dark nights (back-transformed log counts/h: control, mean = 3.63, lower = 3.62, upper = 3.64; experimental, mean = 4.47, lower = 4.37, upper = 4.56; LMM, pMCMC < 0.001, Table 2 and Additional file 1: Figure S1b), but these differences depended on time of day. While birds under both light treatments showed similar activity levels from noon until the evening hours, they differed in behaviour at night and in the morning (Figure 2b). This was due to the fact that rural birds in the light-at-night group greatly increased activity during these hours. In contrast, urban birds showed lower activity levels in the light-treated group than in the group exposed to darkness at night (Figure 2 and Additional file 1: Figure S1b).

**Table 2:** Variation in mean hourly locomotor activity in winter and summer

	**Winter**				**Summer**			
**Parameters**	**Estimate**	**Lower 95%**	**Upper 95%**	**pMCMC**	**Estimate**	**Lower 95%**	**Upper 95%**	**pMCMC**
intercept	0.44	0.35	0.53	< 0.001	0.63	0.50	0.75	< 0.001
time of day	−0.01	−0.01	0.00	**< 0.001**	−0.01	−0.01	−0.00	**< 0.001**
treatment	0.33	0.22	0.43	**< 0.001**	0.41	0.23	0.59	**< 0.001**
origin	0.03	−0.01	0.13	0.629	0.12	−0.06	0.29	0.168
time of day * treatment					−0.01	−0.02	−0.01	**< 0.001**
time of day * origin					0.01	0.00	0.01	**0.030**
treatment * origin					−0.46	−0.71	−0.21	**< 0.001**
time of day* treatment* origin					0.01	0.00	0.01	**0.007**

Models are LMMs. Reference levels: treatment = control group, origin = rural birds. For each parameter we show the estimated mean, the lower and upper 95% CI and the p-value calculated based on MCMC (pMCMC). Non-significant interactions were sequentially removed (empty cells). Significant results are printed in bold.

**Figure 3:**
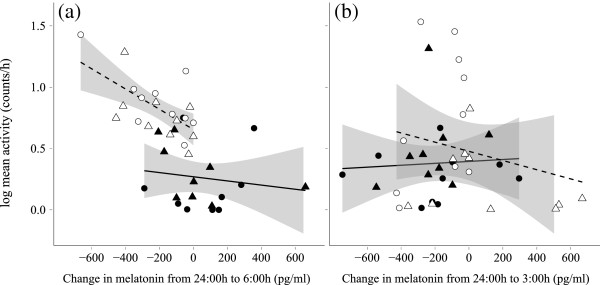
**Relationship between melatonin and dawn activity.** X-axis represents change in melatonin between midnight and either 6 am (winter, **(a)**) or 3 am (summer, **(b)**), Y-axis represents log-transformed mean activity counts in the hour preceding the onset of morning civil twilight (winter: 6:45, summer: 3:45). Dashed (light-at-night) and solid (dark night) black lines represent regression slopes from fitted linear models. Grey shaded areas represent 95% CI. For sample sizes and meaning of colours and symbols see Figure 1.

Most birds showed the general pattern of a drop in melatonin from midnight to morning hours. However, we also observed an occasional increase in melatonin from night to morning in some individuals, in both winter (control: N = 8, experimental: N = 0) and summer (control: N = 3, experimental: N = 4) (Figure 3). To examine the relationship between melatonin and activity patterns, we related the difference between midnight and morning concentrations to the birds’ mean activity in the hour preceding civil twilight. In the winter, the change in plasma melatonin concentration from night to morning was significantly related to locomotor activity in the morning, but only in the light-at-night group (LM, R^2^ = 0.67, F_4,34_ = 17.05; interaction treatment*change in melatonin: P = 0.009, Additional file 1: Table S3, Figure 3). In the summer, there was no significant relationship between change in melatonin and activity in the hour before morning twilight (LM, R^2^ = 0.26, F_4,34_ = 2.95; P = 0.73, Additional file 1: Table S3, Figure 3).

## Discussion

Based on data from light-loggers on free-ranging urban European blackbirds [13] we simulated light-at-night exposure in the laboratory and found that it led to reductions in melatonin secretion, which depended on the time of year and time of night (Figure 1). In particular, in winter (Figure 1a) plasma melatonin concentrations were reduced in the beginning and at the end of the night, around the time of evening and morning twilight. In summer, we detected an overall significant main effect of the light-at-night treatment, as melatonin concentrations were lower in the experimental group during the whole night. Overall, our results suggest that light-at-night of very low intensity is able to reduce melatonin release in blackbirds. We speculate that light-induced melatonin suppression may lead to an altered perception of daylength in this species, in particular in winter (late January), when the birds’ reproductive system is reactivated under the influence of increasing daylength [55]. Indeed, in winter the suppression of melatonin in the late night/early morning correlated with the amount of activity just preceding dawn in the birds exposed to light-at-night (Figure 2a): the greater the reduction in melatonin, the greater the amount of morning activity (Figure 3a). The differences in early morning activity could be particularly relevant for the blackbirds’ breeding biology, because at this time the birds’ song activity and mating activity peaks [12,56].

Suppressed melatonin and increased activity may suggest a physiological mechanism underlying the advanced onset of morning activity and song recorded in several urban bird species in late winter and spring [11,12,54,57]. Based on both, a shorter duration of elevated melatonin and of restfulness at night, blackbirds appeared to have indeed interpreted an urban-like illuminated night as being shorter than a dark night [13]. The findings of an advanced activity onset of birds exposed to light-at-night fit well with considerations from early chronobiology that were based on oscillator theory [58,59]. In addition to its possible, direct effects on melatonin, nocturnal illumination could also affect the circadian system because it modifies the properties of the diel light signal. The diel light signal is the most powerful synchronizing cue (*Zeitgeber*) of the circadian system. Light-at-night weakens the strength of the *Zeitgeber* (given by its amplitude [58]) because the difference between the darkest and brightest illumination over the course of a day decreases. Using songbirds as study subjects, Aschoff and Wever [59] have shown that such a decrease in *Zeitgeber* strength advanced the timing of activity (i.e., the phase angle difference, see also [60]). Similarly, the differences in melatonin amplitude between summer and winter seasons are also in keeping with early chronobiological observations of changes in the circadian system associated with seasonal change in the *Zeitgeber* signal [60,61].

Our findings may have important implications for understanding the control of seasonal processes in urbanized birds, because advanced timing of reproduction has been reported in many species in cities [62,63]. In particular, European blackbirds from an urban population showed earlier gonadal growth than rural counterparts [55], and this has been recently linked to the exposure to light-at-night [13]. Could the reduced release of nocturnal melatonin due to light-at-night, as observed in the present study, explain the advanced reproductive physiology of urban birds? Although the direct role of melatonin in photoperiodic time measurement in birds has been long debated [15,64], new evidence suggests that melatonin can affect some seasonal processes. For example, exogenous melatonin administration can down-regulate the volume of two brain areas involved in seasonal song production, HVC and area X [36]. In addition, melatonin up-regulates GnIH in both the brain [37] and the gonads [65] before breeding, thus keeping gonadal sizes and testosterone levels low. We suggest that the decrease in melatonin as a consequence of light-at-night in winter may provide animals with the physiological signal that the night is shorter than the actual photoperiod, and therefore speeds up reproductive activation. We have recently shown that the blackbirds subjected to 0.3 lux of light-at-night developed the reproductive system almost a month earlier than birds kept under dark nights [13]. However, in this former study we found differences in the response of urban and rural blackbirds. Light-at-night had a stronger, accelerating effect on the termination of reproduction in late spring in urban compared to rural birds. In the present study, however, urban and rural birds did not differ in the melatonin response to the night-light stimulation in both seasons. Thus, the melatonin findings reported here can explain overall effects of night-at-light, but do not elucidate differences between urban and rural blackbirds in their gonadal responses to nocturnal illumination. For these differences, it seems that other major factors may be involved. Interestingly, the only interaction between origin and treatment detected in the present study was found for the summer and concerned activity levels. Light at night suppressed activity in urban birds, while it activated rural birds (Additional file 1: Figure S1b).

It is worth to notice that the low nocturnal light levels to which birds in our study were experimentally exposed are similar to those which under natural conditions, occur during full moon. Studies on rodents have shown that a full moon night can impair the properties of the circadian clock and the phase-angle relationship with the external light–dark cycle [41,66]. However, full moon does not occur as regularly as night-at-light in cities, and it is unlikely that it will affect seasonal rhythms in terrestrial songbirds. Although a single long day is potentially able to alter the response of the avian reproductive axis [67-69], the downstream effects on the time of reproduction (i.e. lay dates) may be limited [68].

Overall melatonin levels differed seasonally. Both maximum amplitude and average concentration were lower in winter than in summer, irrespective of the light treatment. Seasonal variation in avian melatonin profiles has previously been studied with equivocal results in a number of long-distance migrants and arctic species [35,70-72]. Data on seasonal change in diel melatonin in non-migratory songbirds, to our knowledge, are limited to the house sparrow (*Passer domesticus*) [73]. In this species, melatonin amplitude was found to be higher in spring and summer than in winter. Our results, therefore, confirm and expand previous data on seasonal variation in melatonin amplitude in passerine species, but more experimental work is needed to understand the causes and consequences of such variation.

In additional to seasonal differences, individual amplitudes of the diel melatonin secretion tended to be lower in the experimental birds than in the control individuals (Table 1). Perhaps this trend indicates that light-at-night may not only have the capacity to reduce the duration of nocturnal melatonin concentration, but also its diel amplitude. In migratory songbirds, melatonin amplitude has been found to be functionally related to the amount of nocturnal restlessness, or *Zugunruhe*, such that during times when birds show *Zugunruhe* at night circulating melatonin concentrations decrease [71]. This decrease in melatonin levels at night has been theorized to reduce the degree of self-sustainment of the circadian clock, allowing for alternative temporal activity patterns such as nocturnal migratory activity [29]. We suggest that light-at-night, via melatonin-suppression, may also reduce self-sustainment of clocks in avian urban-dwellers. Recent data from our study populations partially support this hypothesis [74]. Urban birds seem to have faster but also weaker circadian clocks than rural conspecifics, although it is not clear whether these differences have originated from micro-evolutionary changes or from a phenotypic response to different environmental conditions in the city, such as after-effects of light at night [74].

## Conclusions

Light pollution, in the form of artificial outdoor and indoor lighting, has become a public health issue as it is now evident that exposure to light-at-night can promote de-synchronization between the endogenous circadian clock and the natural light/dark cycle, with profound downstream negative effects [2]. Here we show that light-at-night can reduce melatonin release in European blackbirds, and we believe that this finding offers new insights in the mechanisms through which urbanization affects biological rhythms of songbirds, and potentially, of other organisms. Follow-up work is needed to address the possible implications of a change in the duration and amplitude of nocturnal melatonin for properties of the circadian clock and for downstream processes [74]. In addition, there may be other physiological costs arising from light-at-night. For example, it would be interesting to know the sleep patterns of birds under light-at-night. Sleep is an important physiological process whose benefits are well known [75], although the underlying proximate and ultimate causes/mechanisms are still only partially understood [76]. Given our results of shortened duration of elevated melatonin and increased activity levels, we hypothesize that light-at-night may disrupt sleep, with potentially serious health consequences [75,77]. Furthermore, night-time light exposure may disrupt physiology through other mechanisms such as alterations in corticosterone rhythmicity or clock genes, as recently shown in rodents [78]. Taking this all together, we call for an improved understanding of the potential physiological costs of the reduction in melatonin levels caused by light-at-night.

## Materials and methods

### Animals

Between May and July 2010 we caught adult male European blackbirds (urban = 20, rural = 20) with mist-nets set up at dawn in the city of Munich, in southeast Germany (48° 07’ N, 11° 34’ E; 518 m ASL) and in a rural forest near the village of Raisting (47° 53’ N, 11° 04’ E, 553 m ASL), 40 km southwest of Munich. Birds were placed in cloth cages and transported to our facilities in Radolfzell (47° 44’ N, 8° 58’ E, 404 m asl) where they were initially housed in outdoor aviaries. On November 26^th^, 2010, birds were moved indoors into individual cages (width × height × length: 45 × 70 × 80 cm) in two separate rooms. Each room contained 10 city and 10 forest birds, all being initially exposed to light/dark (LD) cycles that simulated the natural variation of photoperiod in Radolfzell, with ~ 0.0001 lux of light at night. One urban bird died on April 1^st^, 2011. Birds could hear but not see each other. Food (Granvit, Chemi-Vit, Italy) and drinking water were available ad libitum. All the experimental procedures were carried out in accordance with the guidelines of the Department 35 of the Regional Commission Freiburg, Baden Württemberg, Germany. We used the same animals and the experimental set-up described below to test different hypotheses about the effects of light-at-night on daily and seasonal cycles of blackbirds [13]. Specifically, data on the link between light-at-night and timing of reproduction and molt provide a valuable and unique background for the interpretation of the results of this manuscript [13].

### Light treatment

The experiment started on December 18^th^, 2010. Photoperiod followed the local natural variation of daylength in both treatment groups throughout the experiment. All birds stayed under a light/dark (LD) cycle. Control birds were exposed to very low light intensity at night, whereas experimental birds were exposed to dim light-at-night (see below). Daytime light intensity in both groups ranged from 250 to 1250 lux within each cage, and was provided by dimmable fluorescent white bulbs (Biolux 36 W, Osram, Germany) emitting light at wavelengths covering the human visible spectrum. Because lowest light intensity of dimmable fluorescent bulbs was still very high (~ 20 lux), we used a dimmable incandescent light bulb (SLV Elektronic, Germany, wavelength 450–950 nm) to simulate the low light intensities which free-roaming blackbirds experienced at night [13]. We chose this type of light bulb because it is a common light source in urban areas, e.g. for outdoor light decoration of houses, and is representative of the spectrum that street lights deployed in the city of Munich emit (yellow-red lights). Light intensity at night in the experimental group was set at 0.3 lux, and represents the mean of measurements at all four perches. Control birds were exposed to a light intensity of ~ 0.0001 lux during the night, provided by the same light bulb type as that used for the experimental group. This light intensity of ~ 0.0001 lux was used to allow the birds to orientate in the cage at night. Each group was exposed to a twilight phase of 35 minutes both in the morning and evening. Light programs of both rooms were controlled by Gira Homeserver (Germany). Light intensity in the cages was quantified at all four perches in a cage by using a LI-1400 data logger and LI-210 photometric sensor.

### Melatonin assay

To determine plasma melatonin concentrations, we took blood samples during the periods of July 25–29, 2011, and January 25–29, 2012. Each individual was sampled at four different times of the day during a period of six days. Samples were taken at least 24 h apart from each other to minimize the stress for the animals. The four samples were taken at the following times: at 12:00 (both in summer and winter), 30 minutes after the end of evening twilight (summer: 21:00, winter: 18:00), 24:00 (both summer and winter), and 45 minutes before the start of morning twilight (summer: 3:00, winter 6:00). All reported times are expressed as Greenwich Mean Time (GMT) + 1 h. Each sampling session lasted for a maximum of 15 minutes, for all birds combined. We used headlamps with dim red light to catch and bleed the birds. Birds were bled outside the experimental rooms to minimize disturbance and stress to the other animals. We punctured the brachial vein with a 25-g needle and collected 200 μl of blood in a hematocrit capillary. Blood was immediately stored on ice. Plasma was separated from red blood cells by centrifugation within 30 minutes after the end of the sampling and then stored at −80°C. The concentration of plasma melatonin was measured by Radioimmunoassay (RIA) [79], which we validated for European blackbirds (Additional file 1: Figure S2). Dilutions of 4 night plasma pools from blackbirds were parallel to dilutions of the melatonin standard, indicating that there were no matrix effects. Further, we spiked 4 daytime samples that were close to or below the detection limit with 20 pg of the melatonin standard. On average we recovered 24.5 ± 3.25 pg (mean ± SD), suggesting that the assay slightly overestimated the melatonin concentration in the samples. For our analyses we ran four assays. All samples from one individual were included in one assay to reduce inter-assay variation. The inter-assay coefficient of variation was 6.9%, while the average intra-assay coefficient of variation was 4.7% + 3.7% (mean ± SD).

### Activity recordings

Locomotor activity was recorded continuously for the entire duration of the experiment through a passive infrared sensor mounted on each cage (Intellisense, CK Systems, Eindhoven, The Netherlands). Movements were counted and stored as two minute bins on a computer. For the purpose of this study we analysed activity data recorded for seven days prior to each blood sampling session (summer 2011: July 18–24; winter 2012: January 18–24). For each individual we calculated the number of activity counts for each hour of the 24-hr day, averaged for the seven days of recording.

### Statistical analysis

Analyses were conducted with the software R 2.15.0 [80]. All tests were two-tailed and we applied a significance level α = 0.05. All explanatory continuous variables were centred and standardized to facilitate interpretation of the estimates. In all models described we used a Gaussian error structure because they met the assumptions of normality of residuals and homogeneity of variance. If non-significant interactions were present, they were sequentially removed. When linear mixed models (LMMs) were used, individuals were always included as random intercepts to account for non-independency of repeated measures. In these type of models we assessed the significance of model parameters using a Monte Carlo Markov Chain (MCMC) approach through the function *pvals.fnc* in the R package *languageR*[81]. P-values (pMCMC) were calculated based on the posterior distribution of model parameters (50000 iterations). When significant interactions were detected, we evaluated them running independent linear models at each time of day (Additional file 1: Table S2).

We analysed the variation in absolute melatonin concentration with LMMs. Log-transformed melatonin concentration was the response variable. We first ran a preliminary model with treatment (dark night/light-at-night), origin (urban/rural), time of day and season as fixed effects. This was done to analyse variation in melatonin concentration between seasons. However, it would have been conceptually misleading to include interaction between time of day and any of the other fixed factors, since the sampling times varied between seasons. Therefore, we ran in-depth models separately for summer and winter, including treatment, origin, time of day, 2^nd^ order polynomial time of day and all their interactions as fixed effects.

In many studies amplitude of the melatonin cycle is estimated from daily variation in melatonin concentration [82,83]. However, in our study we collected repeated samples from individuals and were therefore able to derive a more accurate, individual-based estimate of melatonin amplitude. To take account of the fact that birds may differ in melatonin levels, we calculated amplitude as the difference between the maximum and minimum daily melatonin concentrations for each individual. This parameter was then log-transformed and modelled as response variable in the LMM which we used for analysis. Season, origin, treatment, and their interaction were included as fixed effects.

We used LMMs to test for differences in activity levels across treatment groups and origin. Log-transformed mean activity was included as response variable. We first included season as fixed effect, too, but the model did not converge, so we ran two separated models for the winter and the summer. Time of day, treatment, origin, and their interactions were modelled as fixed effects.

Finally, to test the relationship between activity onset and the morning drop in melatonin, we calculated the change in melatonin concentration from night (midnight) to morning (summer: 3 am; winter: 6 am). To reduce bias due to different mean daily melatonin concentrations in different birds, these changes were calculated on an individual basis. We used linear models to analyze log-transformed mean activity levels during the hour preceding morning twilight to treatment in relation to origin and change in melatonin level (modelled as fixed effects).

## Abbreviations

GnIH: Gonadotropin-inhibitory hormone; LD: Light/dark cycle; LM: Linear model; LMM: Linear mixed model; MCMC: Monte Carlo Markov Chain; CI: Confidence intervals; SD: Standard deviation; SEM: Standard error of the mean.

## Competing interests

The authors declare that they have no competing interests.

## Authors’ contributions

DMD and JP conceived the study. DMD, WG, BH, and JP designed the experimental set-up. DMD and JP executed the experiments. DMD analysed the data and wrote the first draft of the manuscript. All authors read and approved the final manuscript.

## Supplementary Material

Additional file 1 **Figure S1.** Effect of light at night on 24-hr locomotor activity levels of blackbirds. Activity was monitored through infrared sensors mounted on top of each bird’s cage. Error bars depict s.e.m. For statistical specifications see Materials and methods section and Table S3. **Figure S2.** Validation of the RIA assay for the European blackbird. We used additional day- (N = 8) and nighttime (N =8) samples from different individuals. The diluted plasma samples from our blackbirds (open symbols) are parallel to the dilutions of the melatonin standard (black dots), indicating that there are no matrix effects. **Table S1.** Variation in individual amplitude of plasma melatonin concentration. Amplitude was calculated as the difference between the minimum and maximum daily value of melatonin concentration for each individual bird. Reference levels for season: summer, for treatment: control group, for origin: rural birds. **Table S2.** Post-hoc independent linear models (LMs) for plasma melatonin concentration. LMs were run to test the significant interaction time of day*treatment found in the model for the winter melatonin (see Table S1). Reference levels: treatment = control group, origin = rural birds. **Table S3.** Relationship between melatonin concentration and activity in the morning. The log-transformed average activity in the hour preceding the onset of morning civil twilight was related to the change in melatonin levels between night (midnight) and morning (3 am in summer, 6 am in winter). Models are LMs. Reference for melatonin change is midnight sample, for treatment is control group, for origin is rural birds. Non-significant interactions were removed (empty cells). Click here for file
