# Supplementary material for: Urban-like night illumination reduces melatonin release in European blackbirds (Turdus merula): implications of city life for biological time-keeping of songbirds
Source: Front Zool. 2013 Oct 3;10:60. doi: 10.1186/1742-9994-10-60 (PMC3850952; doi:10.1186/1742-9994-10-60)
Supplement: Additional file 1 — Figure S1. Effect of light at night on 24-hr locomotor activity levels of blackbirds. Activity was monitored through infrared sensors mounted on top of each bird’s cage. Error bars depict s.e.m. For statistical specifications see Materials and methods section and Table S3. Figure S2. Validation of the RIA assay for the European blackbird. We used additional day- (N = 8) and nighttime (N =8) samples from different individuals. The diluted plasma samples from our blackbirds (open symbols) are parallel to the dilutions of the melatonin standard (black dots), indicating that there are no matrix effects. Table S1. Variation in individual amplitude of plasma melatonin concentration. Amplitude was calculated as the difference between the minimum and maximum daily value of melatonin concentration for each individual bird. Reference levels for season: summer, for treatment: control group, for origin: rural birds. Table S2. Post-hoc independent linear models (LMs) for plasma melatonin concentration. LMs were run to test the significant interaction time of day*treatment found in the model for the winter melatonin (see Table S1). Reference levels: treatment = control group, origin = rural birds. Table S3. Relationship between melatonin concentration and activity in the morning. The log-transformed average activity in the hour preceding the onset of morning civil twilight was related to the change in melatonin levels between night (midnight) and morning (3 am in summer, 6 am in winter). Models are LMs. Reference for melatonin change is midnight sample, for treatment is control group, for origin is rural birds. Non-significant interactions were removed (empty cells). [file 1742-9994-10-60-S1.doc]

**Additional file materials**

**Additional file figures**


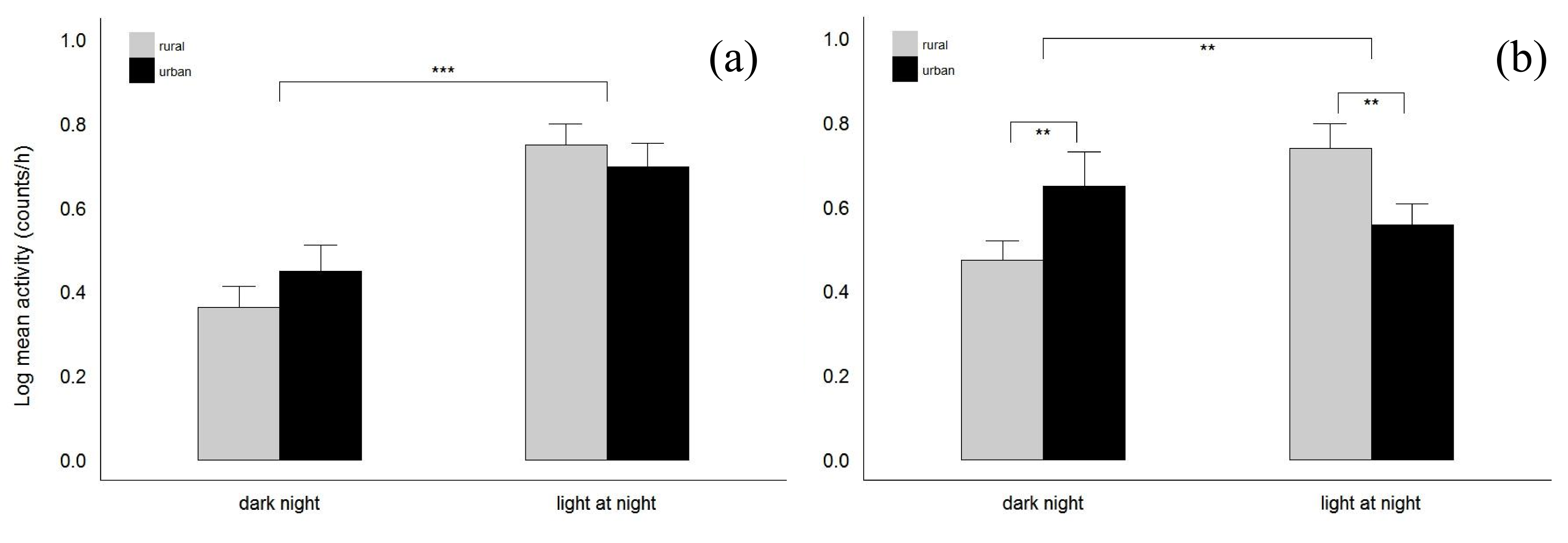


**Additional file 1: Figure S1.** Effect of light at night on 24-hr locomotor activity levels of blackbirds. Activity was monitored through infrared sensors mounted on top of each bird’s cage. In both winter (a) and summer (b) activity levels were significantly higher in birds exposed to light at night than in subjects under dark nights. Furthermore, in both seasons rural birds under dark nights showed less locomotor activity than urban conspecifics. The opposite was true during exposure to light at night, where urban birds showed less locomotor activity than rural conspecifics. This pattern was, however, only significant in summer. Error bars depict s.e.m. For statistical specifications see Methods section and Table S3.


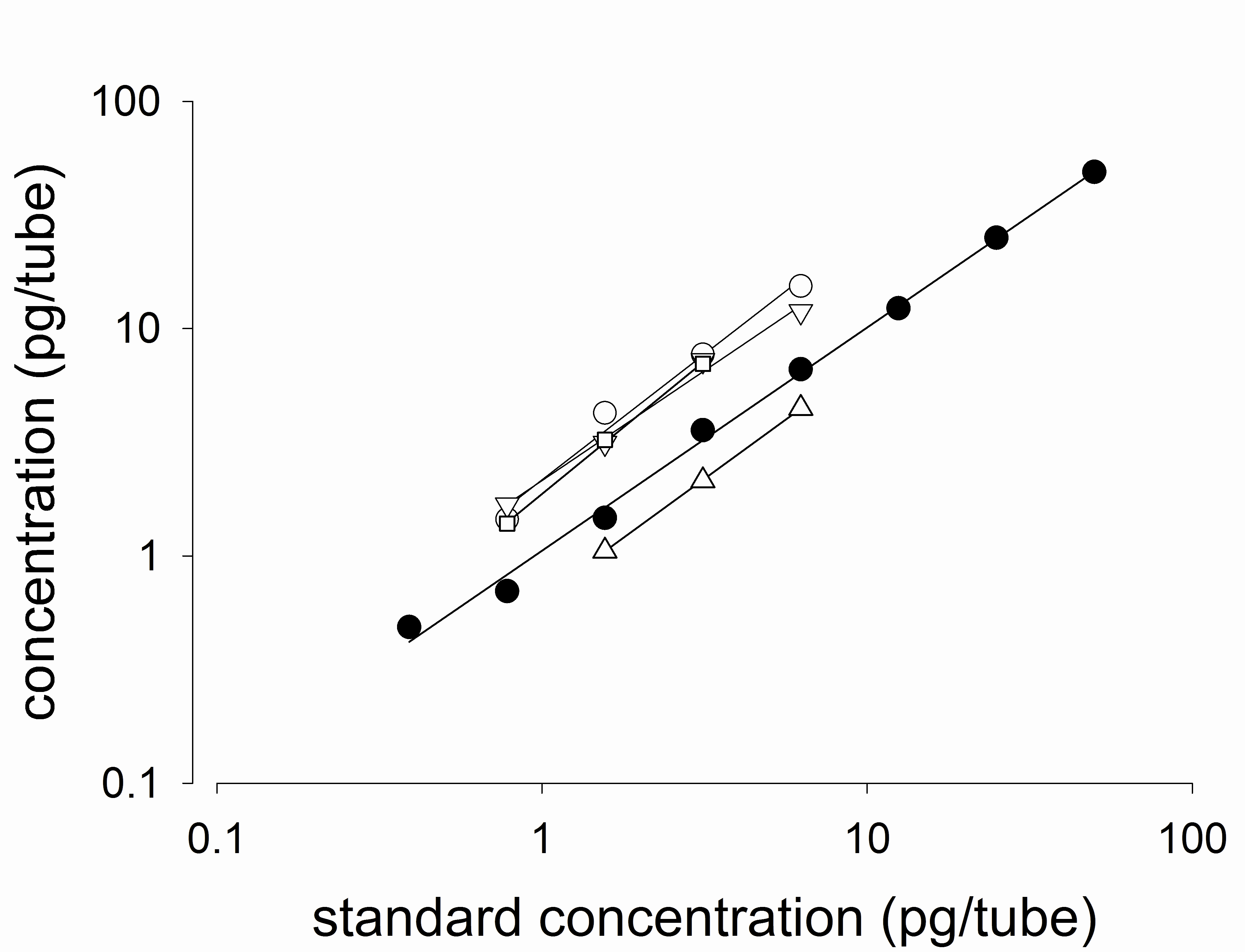


**Additional file 1: Figure S2.** Validation of the RIA assay for the European blackbird. We used additional day- (N = 8) and nighttime (N =8) samples from different individuals to validate the methodology, as it was never been done on this species before. The diluted plasma samples from our blackbirds (open symbols) are parallel to the dilutions of the melatonin standard (black dots), indicating that there are no matrix effects. See Methods section in the main manuscript for more specifications.

**Additional file tables**

**Additional file 1: Table S1**

Variation in individual amplitude of plasma melatonin concentration. Amplitude was calculated as the difference between the minimum and maximum daily value of melatonin concentration for each individual bird. Reference levels for season: summer, for treatment: control group, for origin: rural birds. For each parameter we show the estimated mean, the lower and upper 95 % CI and the p-value calculated based on MCMC (pMCMC).

| **Parameters** | **estimate** | **lower 95%** | **upper 95%** | **pMCMC** |
| --- | --- | --- | --- | --- |
| intercept | 6.22 | 5.80 | 6.67 | < 0.001 |
| season | -0.76 | -1.18 | -0.35 | < 0.001 |
| treatment | -0.43 | -0.87 | 0.01 | 0.052 |
| origin | 0.01 | -0.43 | 0.45 | 0.968 |

**Additional file 1: Table S2**

Post-hoc independent linear models (LMs) for plasma melatonin concentration. LMs were run to test the significant interaction time of day*treatment found in the model for the winter melatonin concentrations (see Table S1). Reference levels: treatment = control group, origin = rural birds. For each parameter we show the estimated mean, the lower and upper 95 % CI and the p-value based on the t-statistic. Significant results are printed in bold.

|  | **mid-day** | | **early night** | | **mid-night** | | **late night** | |
| --- | --- | --- | --- | --- | --- | --- | --- | --- |
| **Parameters** | **estimate** | **P-value** | **estimate** | **P-value** | **estimate** | **P-value** | **estimate** | **P-value** |
| treatment | -0.04 | 0.682 | -0.64 | **0.003** | -0.02 | 0.940 | -1.34 | **< 0.001** |
| origin | 0.01 | 0.938 | -0.05 | 0.802 | 0.07 | 0.806 | -0.06 | 0.832 |

**Additional file 1: Table S3**

Relationship between melatonin concentration and activity in the morning. The log-transformed average activity in the hour preceding the onset of morning civil twilight was related to the change in melatonin levels between night (midnight) and morning (3 am in summer, 6 am in winter). Models are LMs. Reference for melatonin change is midnight sample, for treatment is control group, for origin is rural birds. Non-significant interactions were sequentially removed (empty cells). Significant results are printed in bold.

|  | **winter** | | | | **summer** | | | |
| --- | --- | --- | --- | --- | --- | --- | --- | --- |
| **Parameters** | **estimate** | **s.e.m** | **t** | **P** | **estimate** | **s.e.m** | **t** | **P** |
| intercept | 0.25 | 0.06 | 3.93 | <0.001 | 0.29 | 0.13 | 2.33 | 0.026 |
| change in melatonin | < -0.01 | < 0.01 | -0.47 | 0.641 | < -0.01 | < 0.01 | - 0.35 | 0.731 |
| treatment | 0.41 | 0.01 | 4.21 | **<0.001** | 0.46 | 0.17 | 2.78 | **0.009** |
| origin | 0.01 | 0.07 | 0.13 | 0.908 | 0.21 | 0.17 | 1.24 | 0.225 |
| change in melatonin * treatment | < -0.01 | < 0.01 | 2.76 | **0.009** |  |  |  |  |
| treatment * origin |  |  |  |  | -0.75 | 0.25 | - 2.97 | **0.005** |
